# The Complexities of African Swine Fever Diagnosis and Control in the Developing World: A Narrative Review Focused on Ghana

**DOI:** 10.3390/ani15233448

**Published:** 2025-11-29

**Authors:** Ben Enyetornye, Francis Dogodzi, Aurelle Yondo, Shaun van den Hurk, Kaitlyn Freeman, Jehadi Osei-Bonsu, Patrick Amponsah, Theophilus Odoom, Nicole L. Gottdenker, Binu T. Velayudhan

**Affiliations:** 1 Athens Veterinary Diagnostic Laboratory, College of Veterinary Medicine, University of Georgia, Athens, GA 30602, USA; be96354@uga.edu (B.E.);; 2 Department of Pathology, College of Veterinary Medicine, University of Georgia, Athens, GA 30602, USA; 3 School of Veterinary Medicine, University of Ghana, Legon, Accra P.O. Box LG139, Ghana; fdogodzi@ug.edu.gh (F.D.); theodoom@yahoo.com (T.O.); 4 Joint Graduate School of Veterinary Sciences, Tottori University, Tottori 680-8553, Japan; 5 Livestock and Poultry Research Centre, University of Ghana, Legon, Accra, Ghana; 6 Department of Pathobiology, College of Veterinary Medicine, University of Illinois at Urbana Champaign, Urbana, IL 61802, USA; 7 Kumasi Veterinary Laboratory, Veterinary Services Directorate, Kumasi P.O. Box 1252, Ghana; 8 Accra Veterinary Laboratory, Veterinary Services Directorate, Accra P.O. Box M161, Ghana

**Keywords:** African swine fever (ASF), control, diagnosis, Ghana

## Abstract

African swine fever is a highly contagious and deadly disease of both domestic and wild pigs. In developing countries such as Ghana, the diagnosis and control of ASF are very challenging. In this paper, we discussed factors that account for ASF endemicity in many developing nations, with special focus on Ghana. We identified possible ASF spread via pig value chain through the transportation of live pigs across regions in Ghana. Major factors contributing to ASF spread in Ghana include lack of farmer compensation during ASF outbreaks, free range system of pig farming, porous country borders, lack of rapid on-site diagnostic test kits, unsafe sample collection and transportation to diagnostic laboratories, long diagnostic test turnaround, and improper carcass disposal. We also discuss available diagnostic options for ASF and their limitations. We propose a more holistic approach to ASF control in Ghana. These measures include applying a muti-sectoral approach, rehabilitation of inactive regional laboratories and expansion of services to six newly established regions, promoting point of care testing and developing and implementing farmer compensation plan during outbreaks. These proposed ASF control measures will provide field veterinarians with effective means to make informed decisions while awaiting laboratory confirmation of outbreaks.

## Simple Summary

African swine fever (ASF) is a major disease threat to pig production in many parts of the world, especially in Africa, where the disease is very common. In this review, we discussed diagnostic options, factors, and challenges associated with African swine fever diagnosis and control in developing countries, using Ghana as a case study. We proposed applying a multi-sectoral approach in ASF control in Ghana and the use of rapid diagnostic kits by field veterinarians in order to make informed decisions while awaiting laboratory confirmation.

## 1. Introduction

African swine fever (ASF) is a highly devastating and contagious viral disease of pigs, now ranked as a major threat to pig production globally [1]. African swine fever is caused by a large enveloped virus belonging to the family Asfarviridae [2]. Transmission of ASF virus (ASFV) is complex, as the virus can be transmitted via direct contact between infected and susceptible pigs, fomites, infected ticks, or by consumption of meat from infected pigs or feed contaminated with ASFV [3,4]. Warthogs have been identified as asymptomatic carriers of ASFV [5] in conjunction with *Ornithodoros* ticks, and they are responsible for the sylvatic circulation of ASFV [6], thereby complicating diagnosis and control efforts.

Clinical manifestations of African swine fever vary with factors such as viral virulence, pig breed, exposure route, infectious dose, and endemicity. The disease occurs in peracute, acute, subacute, or chronic forms. Peracute ASF typically causes high fever and sudden death within 1–3 days, often before clinical signs appear. Acute ASF presents with high fever, anorexia, weakness, respiratory distress, and characteristic skin discoloration, with death occurring within 6–15 days depending on strain virulence. Subacute cases show similar but milder signs, with fluctuating fever and pronounced vascular lesions. Chronic ASF, usually linked to attenuated or vaccine-derived strains, develops after 2–3 weeks and is characterized by mild fever, respiratory distress, and joint swelling [7].

The first report of African swine fever was in British East Africa (now Kenya) in 1921. Around the same time, German East Africa, now Tanzania, also reported the outbreak [8]. Approximately 12 years prior to this report, European settlers in Kenya lost a substantial number of domestic pigs to a mysterious disease. For more than thirty years after its discovery, the virus was limited to the Eastern and Southern parts of Africa. By the second half of the 20th century, there were major outbreaks in many African countries [9], including Ghana, where extensive pig production occurs. The first ASF outbreak in Ghana occurred in September 1999, two years after neighboring Togo, Benin, and Nigeria reported outbreaks. While ASF had spread to several European countries, two Caribbean islands, and Brazil, the disease was eradicated from all non-African countries, except the Italian island of Sardinia. However, in 2007, ASF was reported in the Republic of Georgia, with subsequent spread to many neighboring countries. In 2014, it reached the Baltic States and Poland and has become established in wild boar populations, making eradication difficult. In 2018, China reported its first ASF outbreak, and ASF spread rapidly throughout the country to neighboring countries, including the island nations of the Philippines, Indonesia, Timor-Leste, and Papua New Guinea [9].

After the initial 1999 outbreak of ASF in Ghana, within-country disease eradication measures were instituted, followed by intensified surveillance activities which ended in September 2000. Since then, there have been recurrent ASF outbreaks, establishing Ghana an ASF endemic country. During the 2021–2022 ASF outbreak, virus isolates from the Savannah, Greater Accra, Bono East, Ashanti, Central, Oti, and Northern and Eastern regions of Ghana revealed the circulation of two distinct genotypes, the current pandemic strain of ASFV-Georgia/2007 and its derivative strain p72 Genotype II [10,11]. On 1 April 2025, the media reported a suspected case of ASF in Damango (the capital of Savannah region), which killed hundreds of pigs and affected communities such as Attibutu, Boroto, Sori number one, and Canteen [12]. In many countries, ASF negatively impacts the swine industry [3,13,14,15]. So far, it has been reported in 64 countries and territories, accounting for over 934,000 cases in domestic pigs and approximately 31,400 cases in wild boars, resulting in more than 1.99 million pig losses globally [16]. The economic impact has been substantial; for example, ASF outbreaks in the Philippines in 2019 incurred losses estimated at USD 58 million, while a single province in Vietnam reported costs of approximately USD 826,911 in 2020 [17]. In the People’s Republic of China, an estimated cost of USD 111.2 billion was linked to the ASF outbreak in 2019 alone [18,19]. Following the outbreak of ASF in parts of Ghana in the year 1999, an estimated USD 13,323,494 was spent on managing the disease with support from the Food and Agriculture Organization (FAO) of USD 306,000 to help contain its spread [20]. The negative impacts of ASF on the swine industry, especially in low-income countries, are enormous, as they have pushed many swine farmers below the poverty line [21].

Aside from the economic impact, there can be negative emotional and psychological impacts associated with ASF outbreaks [22]. In many African countries, including Ghana, where pigs are used for traditional, cultural, and festive activities, ASF outbreaks can be socially disruptive. Due to limited surveillance in low-resource settings, a proper understanding of ASF epidemiology in the local context in west Africa is usually lacking [23], complicating disease control efforts and leading to transboundary transmission and regional endemicity in most African countries [24].

At present, there is no effective vaccine due to the large genome and complex escape host mechanism mounted by the ASFV [25]. Hence, the only feasible preventive and control measures for ASFV are rapid and accurate diagnosis, implementation of stringent quarantine measures [26], and pig depopulation. However, depopulation of pigs in many developing countries, such as Ghana is very complicated topic because it requires cooperation and coordination between the affected farmers, field veterinarians, diagnostic laboratories, and the government, which is often lacking.

Meanwhile, in many developing and some developed countries, ASF diagnosis and control is challenging due to its similarity to classical swine fever [27] (in countries where it exists) and poor regional coordination between endemic countries and institutions tasked with ASF control [28]. In Ghana, the ASF prevention and control situation is challenging due to complex entanglements of epidemiological, economic, and management factors. In this paper, we discuss the practical challenges associated with ASF diagnosis and control in low to middle income countries, with a special focus on Ghana as a case study and propose an improved framework for ASF surveillance and control.

Ghana serves as an ideal case study because it exemplifies the challenges of African swine fever management in resource-limited settings where recurrent outbreaks occur within a mixed pig industry dominated by smallholder and informal systems, with minimal biosecurity. In addition, the diagnostic capacity is constrained by reliance on centralized laboratories, leading to delays in confirmation. The informal pork markets and porous borders exacerbate the disease spread, while socio-economic dependence on pig farming influences compliance with control measures. Cultural practices and resource limitations further shape management strategies, making Ghana representative of broader patterns across sub-Saharan Africa.

## 2. Methods

This narrative review explored the complexities of African swine fever diagnosis and control in developing countries, with emphasis on Ghana. Literature searches were conducted in Google Scholar, Scopus, and PubMed, without year restrictions and limited to English-language publications. The search terms, combined using Boolean operators, included the following: “African Swine Fever” AND (“diagnosis” OR “diagnostic challenges” OR diagnosis *) AND (“control” OR “control strategies” OR “control measures”) AND (“developing countries” OR Africa OR Ghana) AND (“spread” OR “prevention” OR “One Health” OR transmission). Additional combinations of these keywords were applied to ensure comprehensive coverage. The inclusion criteria were peer-reviewed articles and relevant conference papers addressing ASF diagnosis, epidemiology, and control in developing countries, particularly in Africa. Media reports of ASF outbreaks in various regions in Ghana were included. Studies focused solely on molecular biology without practical diagnostic or control implications, editorials, and non-English publications were excluded. Titles and abstracts were screened, followed by full-text review. Eligible studies were synthesized qualitatively; no meta-analysis was performed, as the review is narrative in nature.

## 3. ASF Spread in Ghana: Possible Role of Pig Value Chain

**Figure 1:**
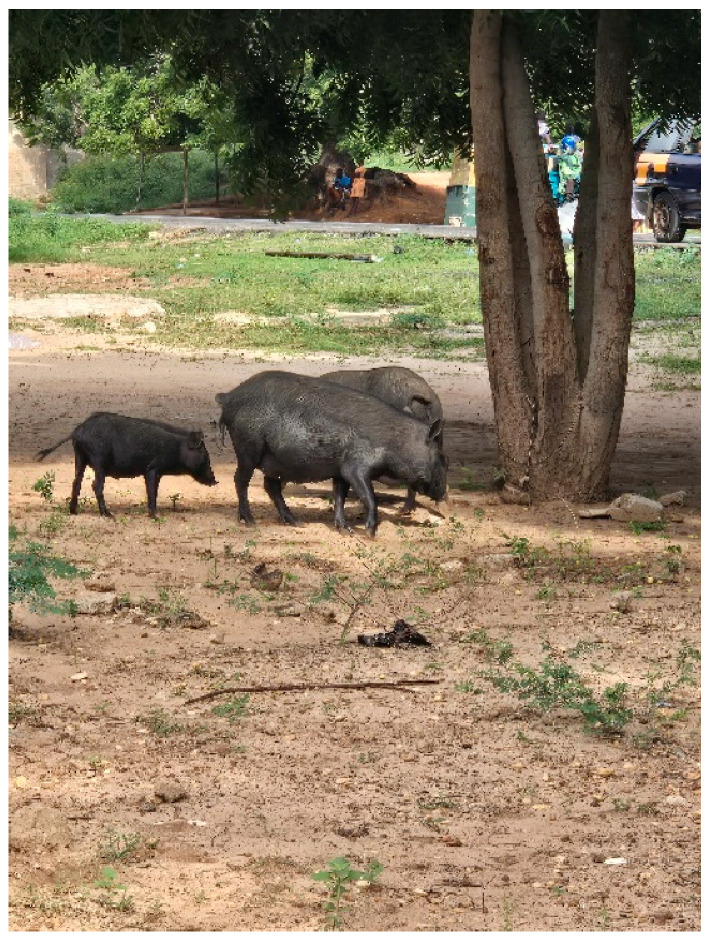
Ashanti black pigs kept under extensive system of pig rearing in a village in Ghana.

The nature and practices within Ghana’s pig value chain significantly contribute to the transmission and persistence of ASF. Generally, across many parts of Ghana, pigs are either kept on farms or in or close to households. When ready for market, ‘small farm’ pigs are either sold directly to individual consumers or to retailers who send them to abattoirs for slaughter and subsequent distribution to restaurants, hotels, individuals, etc. There are also retail businesses that sell grilled, fried, or boiled pork at vantage locations in Ghana [29]. Depending on the size of these retail businesses, owners purchase a few live pigs and keep them in holding pens for daily slaughter or they purchase dressed pigs from other retailers. However, there are geographic difference in pig rearing between the Northern and Southern sectors of Ghana. In the northern regions, pigs (mostly Ashanti black) are predominantly reared on extensive (Figure 1) and semi-intensive mixed farms, where they are allowed to scavenge for food, especially during the dry season [29].

**Figure 2:**
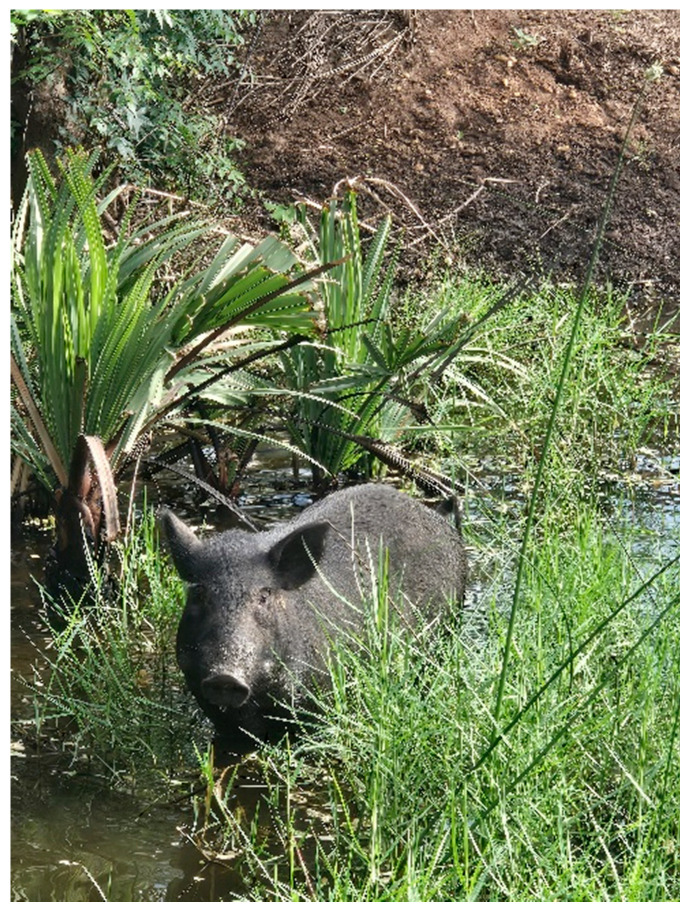
Ashanti black pig roaming in a lagoon in Ghana.

**Figure 3:**
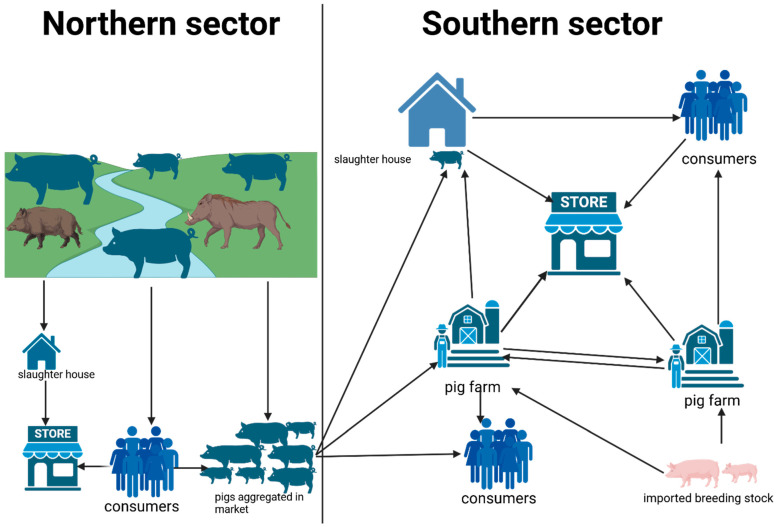
An illustration of the structure of the pig value chain in Northern and Southern parts of Ghana. Ashanti black pigs and sometimes some exotic crosses are commonly transported from the North to the South of Ghana; they interact with wild boar and warthogs, where they may be exposed to ASF prior to movement. Pigs that end up on farms serve as a possible source of ASF, infecting other pigs on the farm. The arrow direction shows the pig or pig products movement pattern.

While scavenging, these pigs move into nearby water bodies (Figure 2) probably to cool off when the sun is hot. This free-ranging behavior often brings them into contact with feral pigs, warthogs, and other wild suids. At the onset of the rainy season in northern Ghana, as communities shift toward crop farming, pigs are considered a nuisance to growing crops and are often sold off. A common practice involves transporting these pigs to the southern parts of the country (Figure 3) [29], typically for slaughter or resale. Unfortunately, this movement is largely informal, occurring without proper veterinary checks, quarantine, or regulation. As a result, pigs potentially incubating or carrying ASF are introduced into new regions.

In southern Ghana, the low cost of these pigs appeals to farmers who purchase them with the intention of fattening and reselling them at a later time. However, the integration of untested animals into existing herds facilitates the silent introduction and spread of ASFV, especially in settings with weak or nonexistent biosecurity measures. Traffic control, an essential component of biosecurity is poorly enforced, allowing vehicles and personnel to move between farms without proper disinfection, further compounding the risk of disease spread. Another critical point of transmission lies in the location of farms near rivers, lagoons, and streams (Figure 2). For instance, in the Ashanti region of Ghana, several pigs farms at Ejisu Juaben, Bosomtwe Atwima Kwanwoma, Kwabre East, and Atwima Nwabiagya are sited close to either a river, stream, or lagoon [30]. Field observations during outbreaks suggest that effluent from decomposing pig carcasses may contaminate upstream water bodies, potentially facilitating downstream transmission and subsequent outbreaks. Our observation is in line with existing reports that identified water as a risk factor for ASF spread [31,32]. When ASF is suspected or confirmed, farmers often panic-sell pigs at steep discounts to avoid total loss, attracting butchers and traders who rapidly move between farms without biosecurity measures, possibly contributing to the virus’s mechanical spread.

## 4. Other Challenges Influencing ASF Epidemiology in Ghana

In many countries, the negative impact of one positive test for ASF can be very devastating for an entire farm, surrounding farms, and farms within that region. It may mean that pigs on farms within a given distance from the affected one may be culled [33], or a strict ban on the movement of pigs and pig products may be enforced. Currently, the Ghanaian government does not have a written policy that promises pig farmer compensation for ASF outbreak-related culls, making farmers reluctant to report suspected cases, contributing to delayed diagnosis and effective ASF control [34]. This situation mirrors challenges in countries such as the Democratic Republic of Congo and Cape Verde, where farmers lack appropriate incentive mechanisms following ASF-related culling, potentially undermining compliance with disease control measures [35]. In the Philippines, for instance, the inadequate adoption of ASF control and monitoring strategies is often reinforced by a lack of financial or institutional incentives [13].

The first ASF outbreak in Ghana (September 1999) was traced to pig farms in Awoshie near Accra and spread to Bawjiase in the Central Region likely caused by the movement of seemingly healthy pigs to “safe” areas. This outbreak ultimately led to the culling of ~6451 pigs, a pig movement ban, and USD 185,000 in compensation to farmers. Prompt identification, aided by prior alerts from neighboring outbreaks, and rapid control measures, including pig movement restrictions, were critical to limiting the spread, although the virulent ASF virus still caused high mortality [19].

**Figure 4:**
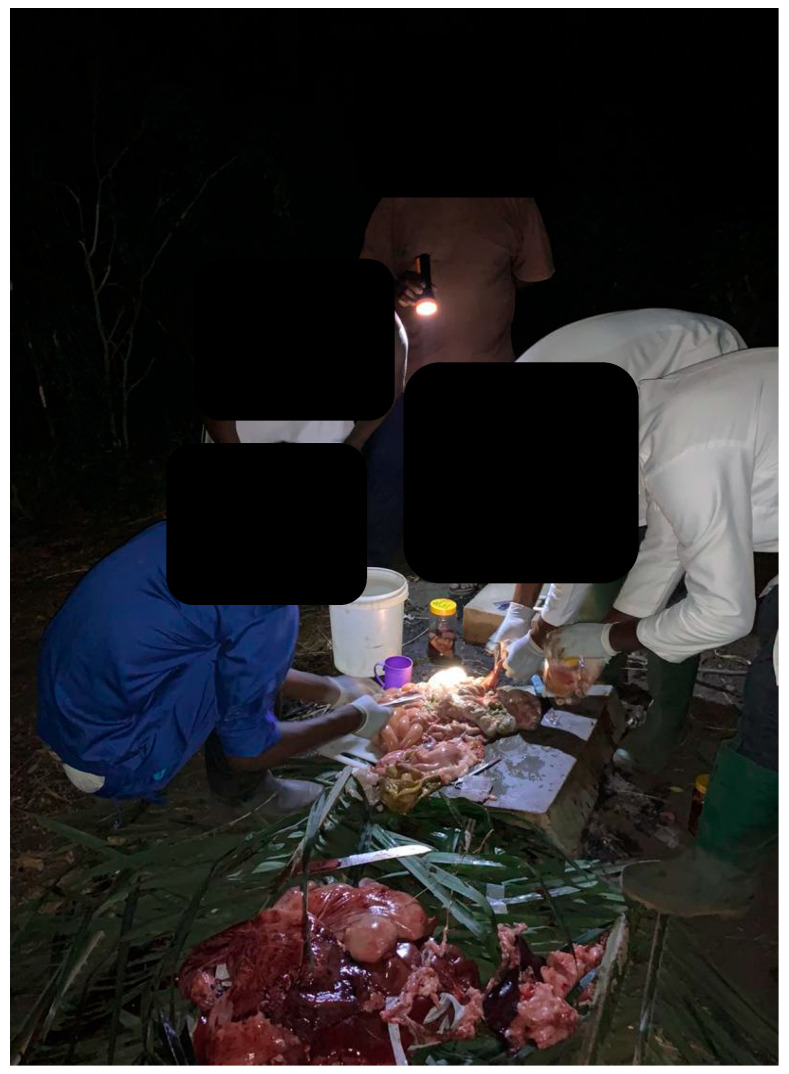
Shows a veterinary team conducting necropsy on palm branches with the farmer holding a torchlight during a light out on the field.

**Figure 5:**
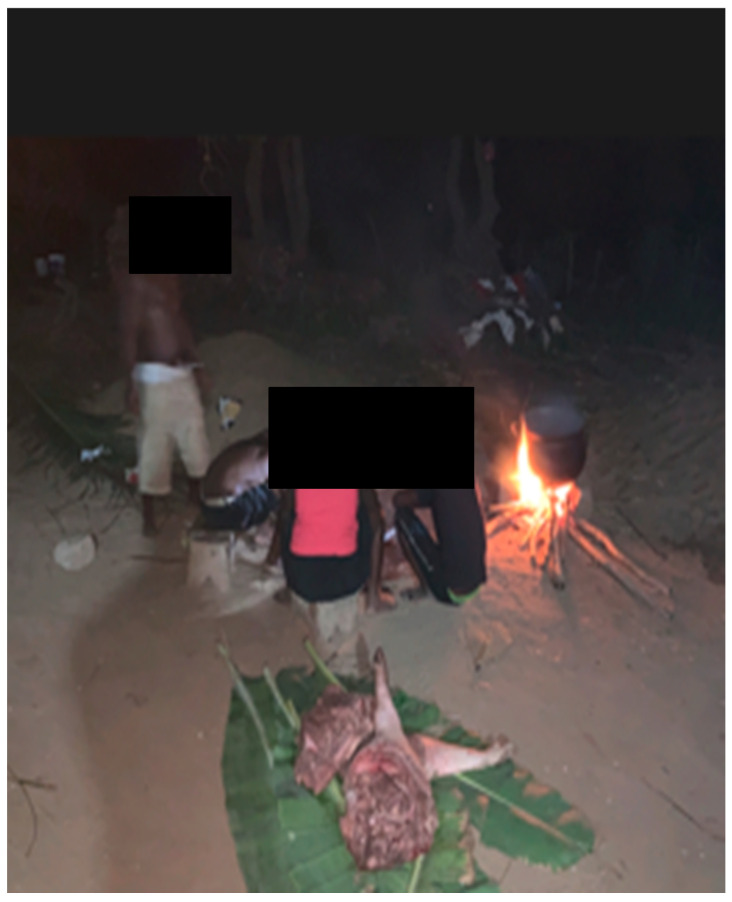
Locals processing suspected ASF carcasses while necropsy was ongoing.

In contrast, no financial incentive was provided during subsequent outbreaks, prompting farmers, looking to protect their livelihoods, to withhold reporting, slaughter pigs quickly, and avoid veterinary involvement [34,36,37]. Some farmers even covered the veterinary sampling costs themselves, only to face depopulation without reimbursement. Field conditions, such as poor lighting during late-night calls (Figure 4), can further hinder veterinarian farm visits to conduct sampling and necropsies. In affected communities, local people may aid the rapid processing of pigs (Figure 5), sometimes interrupting or preventing necropsy and sample collection for ASF testing. Although ASF is notifiable in Ghana [38], the financial burden of sample submission often falls on field veterinarians, discouraging timely reporting and diagnosis.

**Figure 6:**
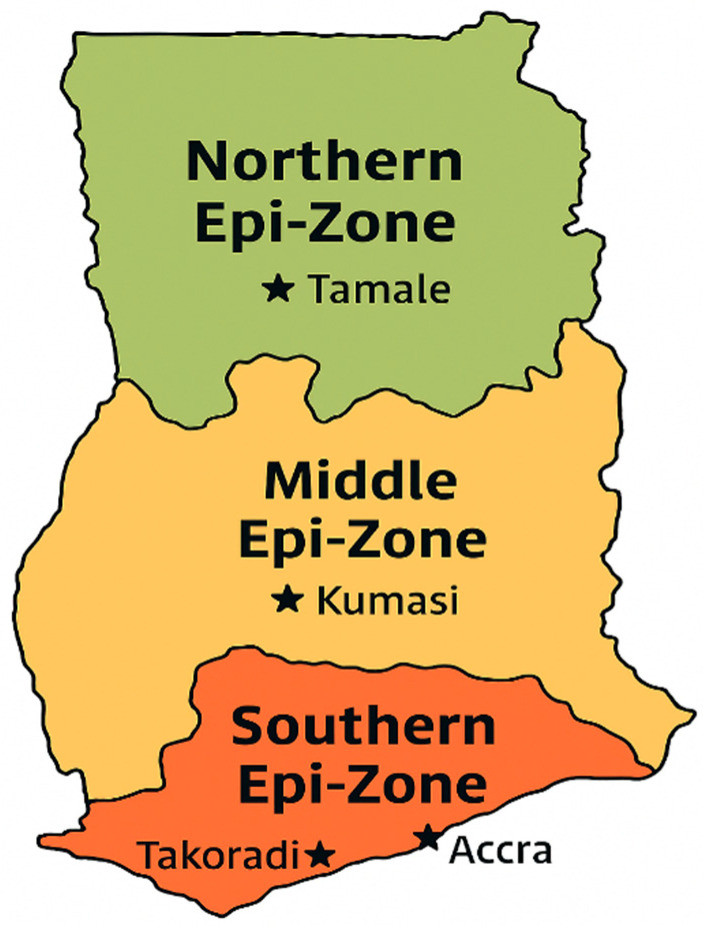
Schematic map of Ghana showing Northern, Middle, and Southern epi-zones.

Efficient ASF diagnosis in many developing countries is hindered by limited diagnostic capacity and the considerable distances between veterinary laboratories and outbreak locations. In Ghana, although the country comprises 16 administrative regions, ASF diagnostic facilities are strategically positioned within three designated epi-zones—Northern, Middle, and Southern (Figure 6)—an arrangement established by the Veterinary Services Directorate to streamline sample submission. However, numerous smallholder farms remain geographically isolated and difficult to access, creating logistical and biosecurity challenges for veterinarians tasked with conducting necropsies, collecting samples, and transporting them across regions for confirmatory testing. For instance, sample transportation [28] can be difficult, with a lack of couriers and vehicles allocated for this. Meanwhile, veterinary laboratories exist in ten of the sixteen administrative regions; however, their functionality is constrained by inadequate equipment, limited and unreliable funding for routine operations, and insufficient technical personnel.

Once samples make it to the diagnostic laboratory, there may be further ‘bottlenecks’ to rapid ASF sample diagnosis in the veterinary diagnostic laboratories, due to high case submission and heavy workloads for laboratory personnel, reagents for common tests like PCR can be prohibitively expensive, and laboratory equipment can be very difficult to maintain and repair. These laboratory issues slowing rapid ASF diagnosis can hinder control efforts, because farmers, who are aware of the implications of a positive test, will quickly resort to rapidly selling off or slaughtering potentially infected pigs to minimize the financial loss. In resource-limited countries, interested organizations such as the Food and Agriculture Organization (FAO), the International Atomic Energy Agency (IAEA), the Defense Threat Reduction Agency (DETRA), and other international donors sometimes provide support ranging from laboratory equipment, vehicles, and reagents to consumables helping in the swift diagnosis of ASF during an outbreak [28,39,40]. Regardless of this aid, inadequate within-country government prevents sustainable supplies of diagnostic reagents and consumables, hindering rapid ASF testing.

The limited number of veterinarians, para veterinarians, and extension officers in the various districts across the country hinders efforts to tackle the myriad animal diseases confronted by farmers [41]. This professional deficit has created the opportunity for imposters posing as vets to further worsen the situation. The lack of farmer education and awareness about the severity of ASF and the scarcity of veterinary professionals are other factors impeding adequate ASF management.

**Figure 7:**
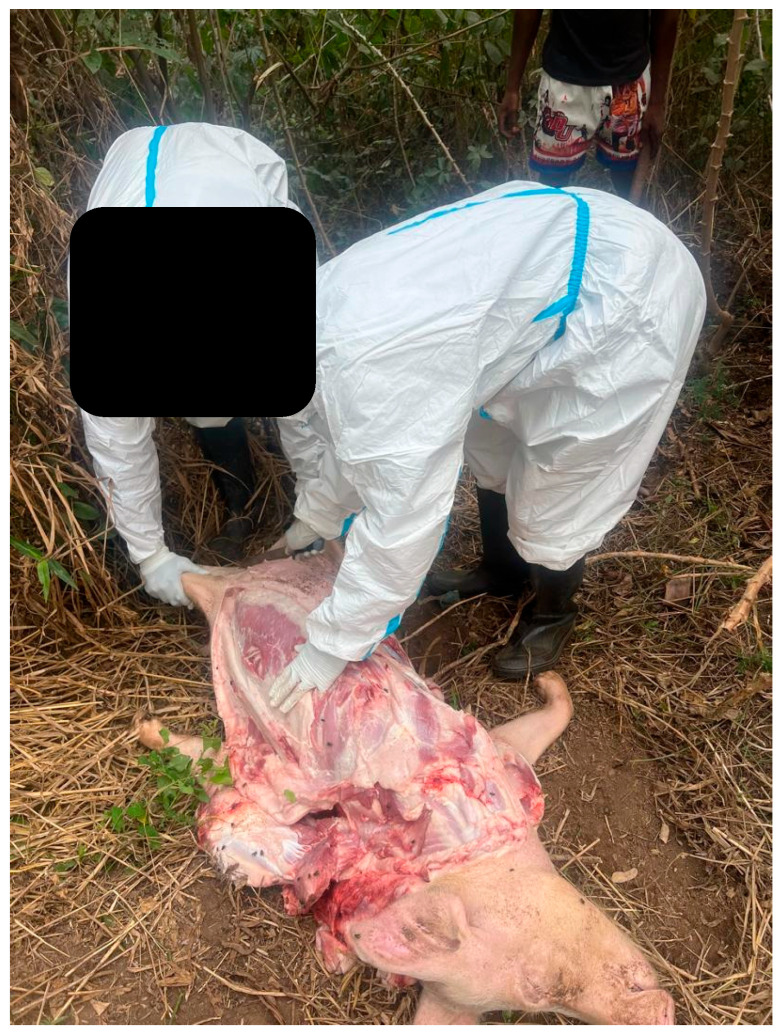
Veterinarians conducting a quick necropsy on suspected ASFV-infected carcass.

**Figure 8:**
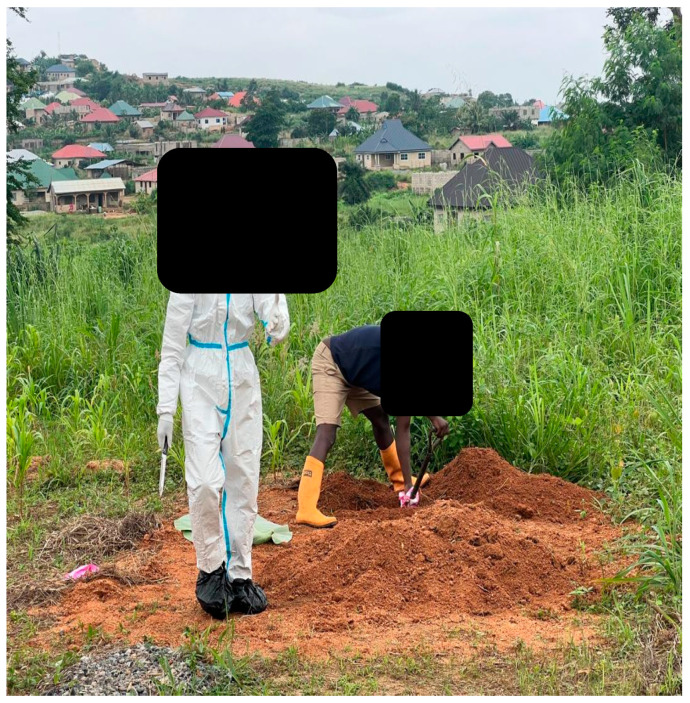
A farm hand digging with a shovel to dispose of suspected ASF-infected carcasses.

In addition, safe and proper carcass disposal, imperative to limit the spread of ASF virus from infected pigs [22,42,43,44] can be challenging. In some instances, on site necropsy (Figure 7), with accompanying shallow burial or garbage dump site disposal of ASF-infected carcasses (Figure 8), allows for spread of the virus via scavengers, such as feral pigs, dogs, and vultures, or can contaminate the environment. There have also been reports of farmers secretly burying and even dumping pigs in the ocean just to avoid culling by the veterinary services [45].

About 56% of smallholder farmers practice the extensive system of pig production, with pigs roaming and scavenging freely in many rural and forest areas in Ghana [46]. This limits biosecurity and increases the ASF risk through exposure to infected materials, other pigs, or sylvatic sources [47]. In regions like northern and coastal Ghana, poor confinement and low biosecurity promote domestic ASF spread via direct contact or fomites, contributing to its high endemicity in Africa, particularly West Africa [48]. Although sylvatic transmission via warthogs and ticks has not been documented in West Africa [9], further research is needed, especially where free-range pigs may contact wild suids [49]. Pigs scavenging for feed, sharing of breeding males, and noncompliance with biosecurity have been identified as risk factors for ASF spread in other African countries like Benin, Nigeria, and Cameroon [50,51,52].

ASF outbreaks in many parts of the world, including Ghana have been linked to live pig movement, pork products, swill feeding, and direct transmission between infected and susceptible pigs [19,38,53,54]. Cross-border and regional movements—common during festivals, funerals, and weddings—facilitate the spread due to porous borders and lack of active surveillance/screening [55,56,57]. A 2002 outbreak likely originated from Togo, about 30 km from Ghana’s border, and spread via the live pig trade to areas like Zabzugu, Ada, and Ashiaman [20,58,59]. Around this time, about 34% of pigs and pork products were traded between Ghana and Ivory Coast [20]. Movement control remains essential for managing contagious diseases like ASF.

For effective ASF control, both passive and active surveillance are important. Unfortunately, synchronized surveillance systems are lacking in many developing countries, including Ghana. Because most pig farms are not registered with the veterinary services or Ministry of Agriculture, there is no proper monitoring unless there are suspicions of ASF or an outbreak occurs before control measures can be put into place.

## 5. African Swine Fever Diagnostic Methods

There are a number of different assays developed for the diagnosis of ASF, and this is an area of active development and research [60,61]. Considering that animal disease diagnosis is rapidly evolving, it is important for veterinary professionals in developing nations to be aware of available diagnostic options and their advantages and limitations in order to make an informed decision based on their prevailing situation. Table 1 summarizes the standard ASF diagnostic methods and assays, along with their advantages and disadvantages. Given the breadth of globally available diagnostic tools for ASF, there is significant potential for Ghana to expand its diagnostic capacity beyond PCR. The integration of validated isothermal amplification techniques and cost-effective rapid tests could substantially enhance early detection and outbreak management, particularly in rural and resource-limited settings.

## 6. Current ASF Diagnostics, Control, and Prevention in Ghana

Laboratory diagnostics play a crucial role in ASF control efforts. In Ghana however, PCR remains the primary laboratory method for ASFV detection, due to its relatively high sensitivity and specificity. However, its reliance on specialized equipment and trained personnel limits its use in rural or field settings. To address this challenge, Ghana has received some international support to build sustainable diagnostic capacity. Despite these efforts, rapid field diagnostics remain underdeveloped.

Since the first ASF outbreak in 1999, Ghana has implemented several control measures, including making ASF a reportable disease, enforcing biohazard safety protocols, implementing strict euthanasia and carcass disposal procedures, conducting passive surveillance, establishing quarantine zones, and imposing swine transport restrictions [38,79]. Suspected ASF cases that test positive result in culling and the establishment of quarantine zones to minimize spread. However, compliance can be difficult when infected pigs are the farmer’s only livelihood, and when, as previously mentioned, government compensation for culled pigs is absent.

Biosecurity, a fundamental preventive strategy promoted in Ghana, involves the disinfection of workers, the use of protective clothing, and the prohibition of swill feeding. Implementation is challenging, especially in rural areas where pigs are kept under free-ranging systems. In farms near forests, where there is increased exposure to wildlife and soft ticks, insect control measures have also been advised [38].

Public awareness and community engagement are part of the current control efforts. Training programs aim to educate farmers on ASF signs, the importance of reporting cases, and basic biosecurity. Collaboration with local veterinarians and extension services supports community-level response. International organizations such as the FAO have contributed technical support for surveillance, diagnostics, and control strategies [80]. Despite ongoing research [81], there is currently no commercial ASF vaccine in use in Ghana [38], although the recombinant live attenuated virus (LAV) vaccine [82] shows promise for a broader range of protection. However, the LAV vaccine shortcomings include potential vaccine viral shedding, infection of pigs by high doses of the live attenuated virus, and the need for strict cold chains, making its use unfeasible on more isolated rural farms.

## 7. One Health Approach for African Swine Fever Control in Ghana

Although ASF is not a zoonotic disease, it has a significant negative impact on food security, rural livelihoods, and the environment [83] (when pig carcasses are buried on farms during an outbreak) in Ghana. The One Health approach, an effective strategy for addressing complex health and environmental challenges through collaborative work among professionals in human and animal health, environmental experts, and other areas of expertise, including policymakers, law enforcement, and farmers [84,85], could be transformative for ASF control strategies in Ghana.

In Ghana, the response to ASF outbreaks is hindered by minimal coordination between veterinarians, public health authorities, and farmers. The absence of collaboration is due to multiple factors emanating from farmers, veterinarians, public health officials, and to a large extent, the Ghanaian government policies. Most farmers fail to report suspected cases due to the fear of having their herds culled and the subsequent financial losses. It would be essential to promote collaboration between veterinarians and farmers based on trust and establish clear communication channels to ensure adherence to surveillance measures, such as reporting suspicious cases.

Many farming activities are conducted in rural and coastal areas [86], where farming represents the primary source of income for local communities. It would also be important for the Ghanaian government to develop policies (such as a compensation scheme for affected farmers) and regulations that take into consideration the socioeconomic realities of pig farmers. Adequate compensation has been identified as a major incentive to promote the honest reporting of suspicious cases of ASF [87].

Additionally, some educational programs promote best practices with the goal of preventing or effectively controlling the spread of viral diseases during ASF outbreaks. These measures include, but are not limited to, prompt identification of sick pigs, good husbandry practices, and an overall enhancement of biosecurity on pig farms. Such educational programs can be championed by regional pig farmer associations with support from the Veterinary Services Directorate and public health personnel in each region of Ghana.

Moreover, veterinarians face many challenges in the absence of adequate diagnostic platforms. Biosafety protocols are largely non-existent, primarily characterized by challenges in proper carcass disposal after necropsies. This poses a risk of possible scavenging by animals and even humans in some instances, contributing to virus spread and the risk of virus persistence and potential host adaptation. It is crucial to raise awareness among the community and inform them of the safety issues, which further emphasizes the need for effective collaboration.

In addition, few studies address how climate affects ASFV transmission in the humid tropical landscapes of Ghana. More research and cross-sector collaboration among veterinarians, farmers, and environmental experts are needed to address this gap, supported by national waste disposal guidelines.

Current ASF interventions in Ghana largely focus on emergency containment, with limited attention to systemic issues like poor waste management, hygiene, and lack of ongoing farmer education. The absence of an enhanced coordinated strategy that incorporates input from farmers, veterinarians, and policy makers ultimately weakens the country’s capacity to effectively manage ASF. Additionally, a system to track all pig farms across the country effectively would be helpful to establish surveillance programs, although this may be undermined by free-ranging pigs. However, it may be a great place to start.

Just recently, the Veterinary Services Directorate developed a Standard Operating Procedure (SOP), aimed at reducing the incidence and impact of ASF in Ghana [88]. Although this initiative represents an important step toward ASF control, it lacks clarity regarding compensation frameworks for farmers whose pigs are culled. This omission could undermine compliance and the overall effectiveness of control measures. A comprehensive well-integrated One Health approach remains the most robust strategy for ASF prevention and control in developing countries

## 8. Operational Framework for the Control and Prevention of ASF in Ghana

Effective implementation of the One Health framework for ASF control in Ghana necessitates the coordinated engagement of multiple stakeholders. The National Emergency Management Committee (NAEMC) functions as the central coordinating body for national animal disease emergency responses. Its membership includes representatives from the Ministry of Food and Agriculture (MoFA), the Veterinary Services Directorate (VSD), the Environmental Protection Agency (EPA), the Information Services Department, the Department of Agriculture, the National Disaster Management Organization (NADMO), the Wildlife Division of the Forestry Commission, pig farmer associations, and other actors within the pig and pork value chain. The committee is chaired by the Chief Veterinary Officer (CVO), who leads the VSD under MoFA.

Within the VSD, the Epidemiology Unit plays a critical role in disease surveillance, diagnostic coordination, and logistics management during outbreaks. At the subnational level, Regional Veterinary Officers (RVOs) and District Veterinary Officers (DVOs) are integral to outbreak response. The RVO oversees regional containment measures, including supervision of field teams, enforcement of quarantine protocols, coordination of logistics, and community engagement. Additionally, the RVO provides weekly reports to the CVO to inform national-level decision-making.

Although Ghana has this established outbreak reporting and control framework—modeled largely on the avian influenza response system—a 2015 study highlighted significant delays in the reporting and management of highly pathogenic avian influenza outbreaks by relevant authorities [89]. This finding underscores the need for not only the development of action plans but also their prompt execution to safeguard both animal and public health.

In practice, the management of animal disease outbreaks in Ghana has predominantly been led by the VSD, with limited engagement from other One Health stakeholders. While this imbalance may reflect resource constraints, adopting a more inclusive multi-sectoral approach would likely improve outcomes. For example, during ASF outbreaks, the Wildlife Division of the Forestry Commission should enhance surveillance of wild suids in the affected areas to identify potential reservoir or spillover populations. Similarly, district and municipal assemblies should enforce restrictions on free-roaming pigs through targeted awareness campaigns and the promotion of confinement practices among farmers.

The VSD must ensure the strict regulation of live pig movements during outbreaks. In exceptional circumstances where movement is permitted, veterinary movement permits should be issued exclusively by qualified veterinarians and verified by law enforcement officers within the relevant districts or regions to prevent the unauthorized transport of potentially infected animals.

Prior to Ghana’s first reported African swine fever (ASF) outbreak in 1999, Côte d’Ivoire—a neighboring West African country—had documented an outbreak in 1996. Subsequently, Togo, Benin, and Nigeria reported outbreaks in 1997 [38,90,91], suggesting potential transboundary transmission of the ASF virus, as proposed by other researchers [92]. These epidemiological patterns highlight the need for strengthened border surveillance and inter-country collaboration in disease monitoring and control.

To reduce the risk of the cross-border transmission of ASF, the Customs Division of the Ghana Immigration Service should strengthen surveillance and border control measures. Strict enforcement of import and export regulations governing pigs and pig-derived products is essential to ensure compliance with national and international biosecurity standards. This includes the systematic monitoring of live pig and pig product movements across borders to prevent the introduction or dissemination of ASF.

The EPA also plays a critical role during ASF outbreaks, particularly in carcass disposal. Given that on-site burial is the most feasible option in many settings and incineration remains cost-prohibitive, the EPA should oversee the burial of carcasses in deep pits located away from areas with high water tables. This practice minimizes the risk of leachate contamination of groundwater and surface water sources, thereby safeguarding both human and animal health.

An integrated response framework that brings together veterinary, environmental, public health, and law enforcement agencies, alongside active community participation, is essential for effective ASF prevention and control in Ghana

## 9. Live Pig Market Value in Ghana and Biosecurity-Linked Compensation Estimation

The prompt reporting of ASF cases is critical for the timely implementation of control measures. To encourage farmers to report suspected cases, it is essential to establish a transparent financial incentive scheme for pigs culled during ASF outbreaks. Linking this plan to compliance with established biosecurity protocols will promote adherence to preventive measures. As a foundation, the development of a biosecurity scoring system (Table 2) tailored to pig farming practices in Ghana is proposed. This system will serve as a practical tool for assessing farm-level biosecurity and guiding compensation eligibility.

To ensure objectivity and minimize evaluator bias, the proposed scoring system may be independently applied by three assessors to evaluate the prevailing biosecurity status of a farm, with the results standardized to a percentage scale to derive the farm’s overall biosecurity score.

With regard to the application of this scoring system to estimate farmer compensation, we propose that farms that have a biosecurity score of >80% (Table 3) should be paid compensation estimated at recovering 70% of the cost of animals culled. Farms that obtain a biosecurity score of 60–79% and 50–59% should be paid compensation aimed at recovering 50–69% and 49–40% of their investment. Any farm whose biosecurity score is below 50% will receive reimbursement aimed at recovering only 25% of their investment. This concept is to encourage the intensive system of pig rearing accompanied with heightened levels of biosecurity, thereby reducing or possibly preventing future ASF outbreaks in Ghana.

Currently, the Livestock and Poultry Research Centre of the University of Ghana, which serves as a primary source of high-quality pig breeding stock and pork, sells breeding pigs at USD 4.0 per kilogram live weight, while pigs intended for meat are sold at USD 3.6 per kilogram live weight. To determine the reimbursement amount for affected farmers, pigs were stratified by age and weight in accordance with the classification standards commonly employed in Ghanaian pig production systems. Weaners are typically between 1 and 2 months of age, weighing 10–19 kg; growers are 3 to 4 months old, weighing 20–40 kg; and finishers are 5 to 6 months old, with body weights ranging from 40 to 50 kg. Generally, pigs are sold upon reaching a live weight of 40–50 kg, while animals maintained beyond this threshold are retained either for breeding purposes or pending market availability. Breeding pigs can weigh between 80 and 150 kg or sometime more, depending on the farm. Based on this information and the proposed biosecurity scoring, we compute compensation (Table 4) for a pig farm with 100 pigs (50 weaners, 20 growers, 20 finishers, and 10 breeding stock,) with a biosecurity score of 80% based on their average weight below.

## 10. Conclusions and Future Directions

Due to the endemic nature of ASF in many developing countries, especially Africa where accurate and prompt diagnosis is a challenge, there is a need for proper coordination between neighboring countries where the disease exists to enhance control efforts. Since ASF is a reportable disease in Ghana, implementing an active surveillance to enhance rapid detection is necessary. ASF outbreaks should be considered a public health threat in Ghana and should be addressed using a One Health approach.

A major barrier to ASF control in Ghana is farmers’ reluctance to pay for costly lab tests. Continuing education through pig farmer associations can encourage prompt reporting, testing, and collaboration with the Veterinary Services Directorate. Although challenging initially, farmers must understand the value of timely ASF diagnosis and the importance of supporting testing services.

Training field veterinarians and para-veterinarians to recognize ASF forms and use point-of-care (POC) diagnostics can enable early screening and biosecurity measures while awaiting lab confirmation. Non-functional regional laboratories should be rehabilitated and equipped to perform PCR diagnostics for the confirmation of African swine fever. For dead pigs, minimally invasive sampling in the field is preferred to full necropsy, which risks spreading the virus to nearby farms. Additionally, zoning of pig-producing areas by ASF status, as practiced elsewhere, could help contain outbreaks.

Ghana currently collaborates with the United States Department of Agriculture, Canadian Food Inspection Agency, and International Atomic Energy Agency to sequence local ASFV strains. These efforts should be expanded regionally to clarify the molecular epidemiology. In regions with limited lab access, wider use of POC testing can support control efforts while samples are processed. Finally, the lack of compensation during outbreaks remains a key factor in the underreporting and spread of ASF. Government action, in consultation with the Veterinary Services Directorate and pig farmer associations, is needed to address this gap and ensure efficient prevention and control of ASF transmission in Ghana.

## Figures and Tables

**Table 1:** Summary of ASFV diagnostic tests.

Type of Test	Advantages	Disadvantages	References
qPCR (Quantitative PCR)	High sensitivity and specificity; rapid turnaround; suitable for multiple sample types; high throughput	Requires specialized equipment; cost-intensive	[28,60,62,63]
Conventional PCR	Previously widely used; simpler protocol	Less sensitive than qPCR; largely replaced in practice	[62,64,65]
Multiplex PCR	Simultaneous detection of multiple pathogens; useful with non-specific signs	More complex assay design and interpretation	[66,67]
Droplet Digital PCR (ddPCR)	High precision; tolerates inhibitors; useful for quantifying viral load	Very expensive; requires specialized equipment	[68]
RPA/RAA(Isothermal)	Portable; fast (under 20 min); highly sensitive	Limited availability; needs further standardization	[69,70]
LAMP (Isothermal)	Minimal equipment; results in 30–60 min; can pair with lateral flow strips	May produce false positives due to primer–dimer issues	[71,72,73]
CRISPR-Cas12a-Based Assays	Very high sensitivity and specificity; field-ready formats like paper strips; broad genotype detection	Not yet widely implemented or validated in low-resource settings	[74,75,76]
Antibody ELISA	Low cost; good for herd-level surveillance; relatively simple	Less useful for early detection; delayed antibody response in acute infections	[28,61,62]
Antigen ELISA	Fast and scalable for herd screening	Lower sensitivity than PCR; often requires confirmatory testing	[62]
Lateral Flow Assays (LFAs)	Portable and visual; no equipment needed; useful for preliminary field screening	Reduced sensitivity and specificity; results should be confirmed with molecular tests	[60,77,78]
Virus Isolation	Confirms presence of live virus; definitive diagnosis	Time-consuming (4–7 days); requires BSL-3 facilities and specialized expertise	[60,61,62]
Hemadsorption (HAD)	Confirmatory test using cellular markers	Labor-intensive; same limitations as virus isolation	[60]
Fluorescent Antibody Test (FAT)	Simple to perform; detects antigen in tissue samples	Lower sensitivity in subacute/chronic infections	[60,61]

**Table 2:** Proposed biosecurity scoring system.

Category	Weight (%)	Description
Citing of farm and access control	15%	Isolation from other pig farms of at least 500 m, fencing, controlled entry points (regular use of visitor logbooks, disinfectant footbaths, vehicle wheel dips)
* Overall health management	20%	Regular deworming history, records of regular veterinary visits, sick animal isolation, records of disease reporting to veterinarians, source of breeding stock, proper weight records of pigs on farm at a given time
Housing and general farm sanitation	15%	Clean pens, drainage, disinfection routines, clean farm surroundings
Feed and water	10%	Safe feed storage facility, no swill feeding, clean water source and storage
Waste and carcass disposal	10%	Proper manure handling, deep burial/incineration of pig carcasses
Employee training and hygiene	10%	Use of boots, gloves, hand washing, documented training on biosecurity
Animal movement control	10%	Quarantine of new animals, movement records including permits obtained
Wildlife and pest control	5%	Solid walls or bird proof nettings, rodent guards, sealed feed storage areas
Availability and implementation of a written biosecurity plan	5%	Tailored biosecurity protocol for farm, which is in use

The biosecurity protocol was adapted from previously published sources with modifications [93,94,95]. Component weights were assigned based on our knowledge of biosecurity practices across pig farms in Ghana. Greater weights were assigned to components considered critical for the effective implementation of biosecurity measures within this context. * Ghana currently does not utilize any African swine fever (ASF) vaccines; therefore, vaccination coverage cannot be considered a valid indicator for assessing animal health in the country. In addition, animal weight records are very important, because live pigs are generally sold based on the live animal weight.

**Table 3:** Score ranges and interpretations.

Score Range (%)	Rating	Interpretation
>80%	Very Good	High level of biosecurity that meets expectations
60–79%	Good	Satisfactory level of biosecurity with some areas for improvement
50–59%	Poor	Below expected standard; improvement needed
<50%	Very Bad	Unsatisfactory level of biosecurity; significant improvement required

**Table 4:** Estimated compensation for a 100-pig farm operating at 80% biosecurity.

Pig Category	Number of Pigs	Average Weight (kg)	Total Weight (kg)	Price (USD/kg)	Value (USD)
Weaners	50	14.5	725	3.6	2610
Growers	20	30	600	3.6	2160
Finishers	20	45	900	3.6	3240
Breeding Stock	10	115	1150	4.0	4600
Total	100	–	3375	–	12,610

Based on a biosecurity score of 80%, 70% of the total value was used to determine the estimated compensation, resulting in USD 8827 for a farm with 100 pigs. The current exchange rate is USD 1.0 = GHS 10.99.

## Data Availability

The original contributions presented in the study are included in the article; further inquiries can be directed to the corresponding authors.
